# Neurosociology and Penal Neuroabolitionism: Rethinking Justice With Neuroscience

**DOI:** 10.3389/fsoc.2022.814338

**Published:** 2022-01-25

**Authors:** Diego Borbón

**Affiliations:** ^1^ The Latin American Observatory of Human Rights and Enterprises, NeuroRights Research Group, Universidad Externado de Colombia, Bogotá, Colombia; ^2^ Rizoma and Legal Psychology Research Groups, Universidad Nacional Abierta y a Distancia, Bogotá, Colombia

**Keywords:** neurolaw, free will, neuropsychology, penal abolitionism, prison abolition, social control, neurocriminology, punishment

## Introduction

Penal Neuroabolitionism is a complementary thesis to the sociological abolitionism of Nils Christie, Thomas Mathiesen and Louk Hulsman (Borbón, 2021). This new approach is based on the findings of science, especially neuroscience, to provide new arguments to the abolitionist perspective that criminal law is an illegitimate mechanism of social control. In that sense, it closely approximates neurosociology as a new scope for transdisciplinary social analysis. In this brief opinion, we offer three commentaries for future work: on the neuropsychological effects of prison, on the ability of neuroscience to analyze and prevent criminogenic social factors, and a critical perspective on free will as a narrative to justify criminal law as a mechanism of social control. These considerations invite scholars around the world to study, within the field of neuroscience, the new arguments for penal abolitionism.

## Neurosociology and Penal Abolitionism

The sociological European penal abolitionism led by Christie, Mathiesen and Hulsman denounces the illegitimacy of criminal law, as a sterile and futile way of imposing pain. Abolitionism holds that the criminal justice system is a social problem in itself and the complete abolition of this system is considered to be the only adequate solution (De Folter, 1986). According to Christie, social systems should be built in a way that minimizes the need to impose pain to achieve social control, as the hell created by man through criminal law is avoidable (Christie, 1981). This argument is further supported by the fact that this pain imposed is usually selective, since it historically punishes particularly vulnerable groups of people (Vegh, 2017). In general, sociological abolitionism has created a body of theories to explain the necessary substitution of criminal law for more humane and restorative alternatives (Christie, 1981; Hulsman, 1991; Mathiesen, 2006; Mathiesen, 2015).

In this direction, neurosociology is a new label for thinking about the human brain and its relationship to human interaction and social organization (Franks and Turner, 2013). Indeed, with advances in science, it is possible to glimpse the usefulness of neurosociology as a new scope for transdisciplinary social analysis to provide innovative arguments in favor of the abolitionist thesis. In particular, a transdisciplinary approach is essential when considering individual and social deviant behaviors.

## Adverse Neuropsychological Effects of Imprisonment

Imprisonment is the most widely used form of punishment in contemporary penal systems. In 2021, more than 11.5 million people were incarcerated in prisons worldwide (Fair and Walmsley, 2021). However, multiple authors have exposed the uselessness of imprisonment, except in a few cases of extreme danger, where the prison fulfills a dubious function of “quarantine” or isolation of the offender (Pereboom, 2003; 2019). Thomas Mathiesen (2006) devoted his intellectual effort to show that, when evaluated in terms of the penal system’s stated goals, prison is a complete failure. The empirical evidence shows that the objectives of imprisonment are not achieved for the most part: prisons do not rehabilitate, and they do not deter from future crimes (Davis, 2003; Mathiesen, 2006; Cid, 2009; Cullen et al., 2011; Mathiesen, 2015). Furthermore, neuropsychology can provide significant arguments against generalizing prison sentences to be the main sanction in penal systems.

Research has yielded valuable results on the adverse neuropsychological effects of prison (Nurse, 2003; Huey and Mcnulty, 2005; Haney, 2012; Schnittker et al., 2012; Brinkley-Rubinstein, 2013; Meijers et al., 2015; Constantino et al., 2016; Haney, 2017; Meijers et al., 2018; Edgemon and Clay-Warner, 2019; Piper and Berle, 2019; Reiter et al., 2020). Overall, these findings tend to correlate prison with poorer mental health, as impoverished spaces, punitive practices, and the prison environment are profoundly disadvantageous factors for mental health and general well-being. For example, the study by Meijers et al. (2018), suggests that 3 months of imprisonment may lead to reduced self-control, increased risk taking and significant deterioration in attention.

According to research from the World Health Organization, within prisons the prevalence of mental disorders is significantly higher than in the general population (Durcan and Zwemstra, 2014) and it has also been suggested that there are ten times more individuals with a mental disorder in jail than in mental hospitals in the United States (Torrey et al., 2014; Semenza & Grosholz, 2019). Instead of fulfilling a positive function, the prison impoverished environments could end up violating minimum human rights standards (Ligthart et al., 2019), and even become criminogenic factors (Vieraitis et al., 2007; Cid, 2009; Cullen et al., 2011; Meijers et al., 2018; Wallace and Wang, 2020).

These are significant findings that should be understood as an open invitation for researchers around the world to study the neuropsychological effects of prison. It is possible to advise, as a criminal policy, to start the reduction of imprisonment as a form of social control, while prioritizing restorative, conciliatory, civil, and therapeutic alternatives to punishment, to reach abolition in the long term. In the medium term, the humanization of the prison, the improvement of the infrastructure and the prison service is an effective way to promote mental health (Gabrysch et al., 2020).

## Neurocriminology and Neurosociology

Since the rise of neurolaw with Taylor et al. (1991), scientific evidence has shown that deviant behaviour has a neurobiological basis, and this has intensified judicial interest in the potential application of neuroscience to criminal law (Glenn and Raine, 2014). In line with this, neurocriminology combines multiple factors (Straiton and Lake, 2021) including genetics, parental influences, early life experiences, hormones, psychophysiology, brain structure, brain function, and neuroimaging to understand why certain individuals are driven to break the law (Glenn and Raine, 2014; Anderson, 2021; Straiton and Lake, 2021). Now that we are confident that neuroscience will have important implications for criminal justice systems (Greely and Farahany, 2019), research on the interaction between neuroscience and criminal law is booming.

Neurocriminology has identified structural and functional deficits in frontal, temporal and subcortical regions, as well as verbal, spatial, and executive dysfunction in antisocial behaviour, and these findings are largely supported by neurological studies of brain trauma in antisocial populations (Schug et al., 2015; Bellesi et al., 2019; van Dongen and Franken, 2019; Katzin et al., 2020). However, some criticize neurocriminology studies for being reductionist. Fallin et al. (2018) argue that most neuroscientists obfuscate legitimate social explanations, transforming complex socially situated behaviors into problems of neurocircuitry.

While this is a legitimate concern, we consider that it is possible to promote a collaboration of disciplines, instead of allowing them to become antagonistic towards each other. Understanding deviant behavior is complex, therefore the careful study of such behavior could benefit greatly from more constructive dialog and collaboration between sociological and neuroscientific disciplines (Aharoni et al., 2019).

Sociology has theoretically and empirically studied social facts such as extreme inequality, poverty, marginalization, and exclusion. In this context, neuroscience can explain how these supra-individual factors affect people’s psychological well-being and leave traces on their neural structures. In addition, lack of nutrition, stress, physical and psychological aggression, stigmatization, and other risk factors might produce adverse neuropsychological effects that can interest criminological studies. With this in mind, it is possible to consider prevention programs that avoid the occurrence of problematic situations related to aggressiveness, impulsivity, lack of empathy, stress and others.

The more effective social programs are implemented, the less criminal law will be seen as a bitter necessity used to threaten individuals in a society. In this transition, institutions such as the prison, or the penal system itself, will be gradually replaced by restorative, community, conciliatory, civil, and therapeutic alternatives. This evolution, under the framework of a true abolitionist proposal, should be far from a dangerousness^1^ perspective that would invade the mental privacy of offenders with neurotechnologies for predicting the risk of aggression or recidivism, which would be unnecessary, inaccurate and affect human dignity.

## Free Will as a Narrative for Social Control

At least in continental European criminal law, with deep Italian and German influences, criminal law has been built unequivocally on free will. The German scholar Claus Roxin maintains that there is an agreement that criminal law must start from free will, although free will cannot exactly be demonstrated (Roxin, 1997). Free will is the foundation of the culpability principle that implies a judgement of reproach to a free and conscientious person who could act according to the law but decided to act against it.

However, in recent decades, neuroscience has provided experiments that suggest that free will does not exist (Libet et al., 1983; Haggard and Eimer, 1999; Soon et al., 2008, 2013; Fried et al., 2011). These studies have not been far from controversy, as several academics also argue against the methodology and conclusions from these findings (Trevena and Miller, 2010; Glannon, 2011; Guggisberg and Mottaz, 2013; Mele, 2014). The truth is that, in general, in philosophy of free will there are two dominant positions: compatibilism and incompatibilism. Compatibilism assumes that even if determinism is true, we would still have free will, while incompatibilism,^2^ excludes the possibility that free will exists if determinism is assumed to be true. (McKenna and Pereboom, 2016). It is also interesting to note that, within sociology, there is a strong debate around structure *versus* agency, which could imply that society determines human behavior (Stones, 2015).

In that sense, many scholars have pointed out the great challenges that criminal law must face. Jurists such as Gimbernat (1971) have affirmed that attempting to found criminal law on the unprovable free will is a battle lost beforehand; fighting it can only increase the irritation of empirical scientists. On the other hand, Winfried Hassemer (2011) argued that the consequences of human biology for criminal justice are obvious and that the only advisable approach is to avoid this discussion, or it will be the end of the criminal justice system.

In this brief commentary we will not deal with the question of whether criminal law can exist without free will. Several scholars such as Günther Jakobs (1992) and Morse (2015) have challenged the idea that the free will debate poses a real threat to criminal law. Rather, we want to point out that free will, despite not being a direct object of sociological questioning (Durkheim, 1966), is of interest in a transdisciplinary debate on penal abolitionism. Free will, in addition to being a metaphysically refutable concept, is a functional idea to achieve social control, as it serves as a justification for holding others morally responsible and facilitates punishment (Clark et al., 2014). This is not only applicable to criminal matters, Nietzsche (1968) argued that the idea of free will, despite being false, served as a narrative for Christianity and priests, for punishment, and therefore, for social control and the maintenance of the status quo. From this perspective, under the allegedly false narrative of free will in the penal system, the State ignores the causes of crime by holding the offender responsible and leaving the social structure intact. In that case, prisons would embody a hidden role in the wider field of political domination and general social control, which would be different from prison’s declared objective of disciplining individuals (Foucault, 1977; Garland, 1991).

## Conclusion

The new thesis of Penal Neuroabolitionism can be nourished by scientific findings. If neuroscience can show that voluntary decisions are not in fact consciously taken nor available to a responsible agent, and furthermore, if physics demonstrates that classical determinism and quantum indeterminism are true, then criminal law might lose its foundations. Without free will, it will be possible to stop simplifying criminal phenomenon and instead to scientifically understand the neurobiological, social, and cultural risk factors behind deviant behavior. With these inputs, it will be possible to offer more effective social programs and eliminate archaic concepts of moral or criminal responsibility. In addition, since it is possible to identify the adverse neuropsychological consequences of prisons, rigorous programs of humanization in the medium term, and of abolition in the long term, should be considered. In the future, neuroscience could even be useful to intervene in brains that clearly present severe pathologies related to deviant behavior. Penal Neuroabolitionism is a new approach, to invite researchers and academics to join in collective efforts to determine how we want to shape criminal policies of the future.

## Footnotes

1 We use the concept of dangerousness in the context of expansive proposals of the punitive power of the State under the narrative that there are dangerous individuals who must be arrested without even having committed a crime. In recent years the concept of Neuroprediction has become popular, which uses neurotechnologies to assess the risk of violence, crime, or recidivism. However, these technologies are still limited in accuracy, and are ethically problematic (Tortora et al., 2020).

2 We have argued that Penal Neuroabolitionism as a criminal policy proposal should be based on an adaptation of hard incompatibilism (Borbón, 2021), which implies that our actions are either deterministic or truly random events and both possibilities exclude free will and moral responsibility (Pereboom, 2003). Now, in our vision, we should adopt a theoretical nuance that we have called humanistic incompatibilism, which would imply, in addition to the fact that free will does not exist and that we are not morally and criminally responsible, that human dignity must be a limit to the advancement of science and justice (Borbón, 2021).
